# Do individuals with inflammatory arthritis receive minimally adequate treatment for incident depression and anxiety: A population-based study

**DOI:** 10.1186/s13075-024-03466-8

**Published:** 2025-01-21

**Authors:** Alyssa Howren, Eric C. Sayre, J. Antonio Avina-Zubieta, Joseph H. Puyat, Deborah Da Costa, Hui Xie, Eileen Davidson, Amit Gupta, Mary A. De Vera

**Affiliations:** 1 Faculty of Pharmaceutical Sciences, University of British Columbia, 2405 Wesbrook Mall, Vancouver, BC V6T 1Z3 Canada; 2 Collaboration for Outcomes Research and Evaluation, Vancouver, BC Canada; 3 Arthritis Research Canada, Vancouver, BC Canada; 4 British Columbia Centre on Substance Use, Vancouver, BC Canada; 5 Department of Medicine, Division of Rheumatology, University of British Columbia Faculty of Medicine, Vancouver, BC Canada; 6 Centre for Advancing Health Outcomes, Vancouver, BC Canada; 7 School of Population and Public Health, University of British Columbia Faculty of Medicine, Vancouver, BC Canada; 8 Faculty of Medicine, Department of Medicine, McGill University, Montreal, QC Canada; 9 Faculty of Health Sciences, Simon Fraser University, Burnaby, BC Canada

**Keywords:** Rheumatoid arthritis, Psoriatic arthritis, Ankylosing spondylitis, Depression, Anxiety

## Abstract

**Objectives:**

Describe patterns of pharmacotherapy and psychological treatment and evaluate receipt of minimally adequate treatment for incident depression and anxiety in individuals with inflammatory arthritis (IA).

**Methods:**

We used population-based linked administrative health databases from British Columbia, Canada to evaluate pharmacotherapy and psychological treatments for incident depression and/or anxiety among individuals with IA and without IA (‘IA-free controls’). We defined minimally adequate pharmacotherapy as antidepressant prescriptions filled with ≥ 84 days’ supply and adequate psychological treatment as ≥ 4 counselling/psychotherapy services. Multivariable logistic regression models were used to evaluate the odds of individuals with IA receiving minimally adequate pharmacotherapy and/or psychological treatment compared to IA-free controls.

**Results:**

6,951 (mean age 54.8 ± 18.3 years; 65.5% female) individuals with IA had incident depression and 3,701 (mean age 52.9 ± 16.8 years; 74.3% female) had incident anxiety. Minimally adequate pharmacotherapy and psychological treatment for depression was respectively observed in 50.5% and 19.6% of those with IA, proportions similar to IA-free controls (pharmacotherapy: aOR 1.10, 95% CI 1.00 to 1.21; psychological: aOR 1.07, 95% CI 0.94 to 1.21). Results were similar regarding anxiety treatment. Individuals with IA had a significantly greater likelihood of dispensing ≥ 1 benzodiazepine (anxiety: IA 45.0%, IA-free controls 39.0%, p-value < 0.001) and ≥ 1 tricyclic antidepressant prescription (anxiety: IA 12.8%, IA-free controls 7.8%, p-value < 0.001). Significantly higher average days’ supply of benzodiazepines was observed for IA (anxiety: IA 123.7 days, controls 112.4 days, p-value = 0.003).

**Conclusions:**

A substantial proportion of individuals with IA were not receiving adequate mental health treatment for depression and anxiety, a finding similar for IA-free controls. The undertreatment of mental disorders for people with IA has well-known negative implications for the provision of effective rheumatology care. It remains fundamental to expand publicly funded health care to include mental health services in an effort to address unmet counselling needs.

**Supplementary Information:**

The online version contains supplementary material available at 10.1186/s13075-024-03466-8.

## Background

Inflammatory arthritis (IA), including ankylosing spondylitis (AS), psoriatic arthritis (PsA), and rheumatoid arthritis (RA), is associated with a substantially increased risk for incident depression and anxiety [1–4]. The negative consequences of comorbid psychiatric disorders are primarily rooted in evidence focused on depression in individuals with RA, and include increased health care use, problems with sleep, poorer self-management, increased disease activity, as well as reduced medication adherence and efficacy of biologic and conventional synthetic disease modifying anti-rheumatic drugs [5–7]. As such, it is imperative to understand whether individuals with IA experiencing comorbid depression and/or anxiety are receiving adequate mental health care.

Despite the substantial risk of depression and anxiety in IA, there is limited knowledge of the sequential diagnosis and treatment – whether pharmacologic or psychological – for these psychiatric disorders. This represents a quality-of-care issue considering guidelines for specific types of IA recommend clinician screening for depression and anxiety, as well as referral for treatment, if necessary [8, 9]. A 2016 cross-sectional study of individuals attending a rheumatology outpatient clinic evaluated depression and anxiety using self-assessment questionnaires and reported that antidepressants or anxiolytics were in use by 13% of individuals with depression and 12% with anxiety [10]. Understanding whether gaps in mental health care exist for individuals with IA necessitates using validated case definitions to identify incident mental disorders following an IA diagnosis and an evaluation of both pharmacological and psychological treatment. Therefore, we aimed to describe patterns of pharmacological and psychological treatment and evaluate the receipt of minimally adequate treatment for incident depression and anxiety in individuals with AS, PsA, and RA using linked population-based administrative health data that captures inpatient and outpatient visits as well as dispensed medications.

## Methods

### Data source

Our retrospective cohort study of individuals with AS, PsA, and RA covering the period of January 2, 2000 to March 31, 2018 used administrative health data available from Population Data BC. Population Data BC maintains individual-level and de-identified longitudinal health services data for the population of British Columbia, Canada, and data holdings accessed for this study include: (1) Discharge Abstract Database (DAD) for inpatient visits [11]; (2) Medical Services Plan (MSP) database for outpatient visits (e.g., general practitioners, psychiatrists, rheumatologists) [12]; (3) Vital Statistics File for deaths [13]; and (4) Consolidation File for demographics (e.g., age, sex) [14]. Information on medications dispensed through community pharmacies and hospital outpatient pharmacies from January 1, 1996, onward is captured in the PharmaNet database [15]. All databases are linked by data stewards at Population Data BC using personal health numbers, and subsequently replaced with random depersonalized identifiers for patient anonymity.

### Source population

Persons with AS were identified with the following case definition: ≥2 outpatient physician visits with a diagnostic code for AS (*International Classification of Diseases*,* 9th Revision* [ICD-9]: 720.X) and/or inpatient visits (*International Classification of Diseases*,* 10th Revision* [ICD-10]: M45.X) in the primary position, with visits occurring ≥ 2 months apart and within 2-years [16]. We modified the following case definition for PsA validated by Lee and colleagues [17] to include inpatient visits in addition to the original definition: 1) ≥ 2 outpatient physician visits with diagnostic code for PsA (ICD-9: 696.X), with at least 1 of these visits to a rheumatologist; or 2) 1 inpatient visit with a diagnostic code for PsA (ICD-10: L40.5). Cases of RA were identified using the following case definition: ≥2 physician outpatient visits with a diagnostic code for RA (ICD-9: 714.X) at least 2 months apart and within 5-years [18, 19]. Several exclusion criteria were also applied for RA: (1) no diagnosis of RA coded by a rheumatologist after a non-rheumatologist outpatient visit coded as RA; (2) 2 subsequent outpatient visits with diagnostic codes for AS, PsA, systemic lupus erythematosus, and connective tissue diseases; and (3) no outpatient physician visits with a diagnostic code for RA after five years of follow-up [18, 19]. Individuals were assigned to a diagnosis of AS, PsA, or RA based on the case definition that was first fully met. The date of the second diagnostic code was designated the IA index date for case definitions that were met using ≥ 2 diagnostic codes. A 10-year run-in period without IA was applied to ensure inclusion of incident IA. In addition, the source population included adults (≥ 18 years of age) from the general population without IA (‘IA-free controls’).

### Study sample

We defined incident depression and anxiety (i.e., occurring after IA index date) from the source population using data from the MSP and DAD administrative databases. We applied an ICD-9/10 case definition for depression (ICD-9: 296.2, 296.3, 296.5, 300.4, 309.x, and 311; ICD-10: F20.4, F31.3-F31.5, F32.x, F33.x, F34.1, F41.2, and F43.2) that was previously validated using clinical charts in British Columbia and Alberta, Canada [20]. The case definition required the aforementioned diagnostic codes for depression to occur as either: 1) ≥ 2 outpatient visits within 1 year; or 2) 1 inpatient visit [20]. We then defined incident cases of an anxiety disorders using an ICD-9/10 case definition that was validated against medical records in Manitoba, Canada (300.0, 300.2/F40-F41) [21, 22]. Our analyses identified three diagnostic groups: (1) incident depression; (2) incident anxiety; and (3) incident depression and anxiety. IA and IA-free controls that met the case definition for depression and/or anxiety prior to the IA index date were excluded from their respective analyses. Individuals with IA and IA-free controls identified as having incident depression and/or anxiety were matched (1:1) based on age, sex, and date of incident depression and/or anxiety diagnosis (± 5 years). In addition, the date of depression and anxiety diagnosis for IA-free controls needed to be on or after the IA diagnosis date of their respective match.

### Outcome ascertainment

#### Pharmacotherapy

We used Anatomical Therapeutic Chemical (ATC) classification codes and DIN/PINs in PharmaNet to identify antidepressants (ATC, N06A) and anxiolytics (ATC, N05B) (Supplementary Table 1). We evaluated practitioner specialty codes from the PharmaNet database to determine the type of prescribing physician.

#### Psychological treatment

Fee-item codes from the BC Medical Services Commission Payment Schedule were applied to identify publicly funded psychological treatments including counselling, psychotherapy, psychiatrist visits, GP mental health planning, and telehealth service (Supplementary Table 2) [23]. Inpatient visits from the DAD database were assessed for at least one depression and/or anxiety ICD-9/10 codes occurring in any diagnostic position to derive the following outcomes: (1) all-cause hospitalization; (2) hospitalization for depression; (3) hospitalization for anxiety; and (4) hospitalization for depression and anxiety.

#### Minimally adequate pharmacotherapy and/or psychological treatment

Minimally adequate pharmacotherapy for both depression and anxiety was defined as the dispensation of antidepressants (ATC N06A) with ≥ 84 days of supply within the first year following diagnosis. For depression, this covers the 12-week acute phase of treatment [24–26]. First-line pharmacotherapy for most anxiety disorders is primarily antidepressants, apart from specific phobias for which pharmacotherapy is not indicated [27]. Whereas anxiolytics are often prescribed for initial short-term use and were therefore excluded from our definition of minimally adequate pharmacotherapy [27].

The receipt of minimally adequate psychological treatment is defined as ≥ 4 counselling or psychotherapy sessions within 12-months following the index date for depression or anxiety. The previously outlined rationale for a minimum of four counselling or psychotherapy sessions is based on prior literature assessing minimally adequate care for depression and an acceptable minimum coverage within the publicly funded health care system in British Columbia [25].

Finally, we evaluated the receipt of minimally adequate pharmacotherapy or psychological treatment within the 12-months following incident depression and/or anxiety. These outcome variables are based on previously established definitions for assessing adequate mental health care for depression [25].

### Covariate assessment

For this study, the covariates we evaluated were age, sex, neighbourhood income quintile, residence (urban/rural), and the Charlson-Romano Comorbidity Index to represent comorbidities [28].

### Statistical analysis

We used descriptive statistics to evaluate sociodemographic characteristics of IA and IA-free controls within each of the diagnostic groups (i.e., depression, anxiety, and depression and anxiety). We evaluated patterns of pharmacotherapy and psychological treatment in the 12-months after index date for incident depression and/or anxiety. We observed a 12-month period to ensure difficult to access services in BC were included [25]. IA and IA-free controls were required to have ≥ 12 months of MSP registration data following their index date for depression and/or anxiety. We assessed pharmacotherapy and psychological treatments in terms of the mean number of events and the proportion of individuals with ≥ 1 event over the 12-month observation period. The Wilcoxon rank-sum test and Pearson Chi-squared test/Fisher’s exact test assessed differences in the pattern of pharmacotherapies and psychological treatments between IA and IA-free controls. We evaluated the 12-month mean days of supply for antidepressants and benzodiazepines. We also evaluated proportion days covered (PDC) for antidepressants and benzodiazepine derivatives, which we calculated as number of days covered from initial prescription date to the end of the 12-month observation period.

Multivariable logistic regression models, adjusted for age, sex, comorbidities, income, and residence, were used to evaluate the odds of individuals with IA receiving minimally adequate pharmacological or psychological care for their incident depression or anxiety as compared to IA-free controls. In addition, multivariable models were stratified by sex and type of IA (AS, PsA, and RA). We used SAS statistical software v. 9.4 (SAS Institute, Cary, North Carolina) for all analyses.

### Study conduct

This study was approved by the University of British Columbia Behavioural Research Ethics Board (H18-03737). Access to data provided by the Data Steward(s) is subject to approval but can be requested for research projects through the Data Steward(s) or their designated service providers. All inferences, opinions, and conclusions drawn in this manuscript are those of the authors, and do not reflect the opinions or policies of the Data Steward(s).

## Results

Altogether, 6,951 individuals with IA had incident depression and 3,701 had incident anxiety (see Table 1 for characteristics of study sample and Supplementary Fig. 1 for cohort creation). The respective incidence rates of depression and anxiety for individuals with IA were 4.6 (95% confidence interval [CI] 4.5 to 4.7) and 2.4 (95% CI 2.3 to 2.5) per 1000 person-years.

**Table 1:** Characteristics of individuals with inflammatory arthritis (IA) and IA-free controls with incident depression or anxiety

	Depression		Anxiety	
Characteristic	IA (*n* = 6,951)	IA-free Controls (*n* = 6,951)	IA (*n* = 3,701)	IA-free Controls (*n* = 3,701)
Age, mean (SD)	54.8 (18.3)	54.8 (18.3)	52.9 (16.8)	52.9 (16.8)
Female, n (%)	4555 (65.5)	4555 (65.5)	2751 (74.3)	2751 (74.3)
Type of inflammatory arthritis, n (%)				
Ankylosing spondylitis	792 (11.4)	---	458 (12.4)	---
Psoriatic arthritis	521 (7.5)	---	326 (8.8)	---
Rheumatoid arthritis	5638 (81.1)	---	2917 (78.8)	---
Charlson-Romano comorbidity index, mean (SD)	0.95 (1.09)	0.37 (1.06)	0.93 (1.04)	0.32 (0.90)
Neighbourhood income quintile, n (%)				
Quintile 1	1585 (22.8)	1495 (21.5)	830 (22.4)	786 (21.2)
Quintile 2	1454 (20.9)	1377 (19.8)	792 (21.4)	718 (19.4)
Quintile 3	1363 (19.6)	1337 (19.2)	697 (18.8)	736 (19.9)
Quintile 4	1349 (19.4)	1327 (19.1)	705 (19.1)	761 (20.6)
Quintile 5	1200 (17.3)	1415 (20.4)	677 (18.3)	700 (18.9)
Residence, n (%)				
Urban	5870 (84.5)	6101 (87.8)	3076 (83.1)	3201 (86.5)
Rural	1081 (15.6)	850 (12.2)	625 (16.9)	500 (13.5)

Descriptive statistics were determined for the year prior to IA index date.

*Abbreviations: IA – inflammatory arthritis; SD – standard deviation*

### Depression

#### Pharmacotherapy

Patterns of pharmacotherapy for IA and IA-free controls with depression are shown in Tables 2 and 3. Tricyclic antidepressants were used more often by individuals with IA in terms of the proportion with ≥ 1 tricyclic antidepressant prescription dispensed (IA: 10.8%, IA-free controls: 7.1%, p-value < 0.001). Benzodiazepine derivatives were the main anxiolytic dispensed and 26.0% of individuals with IA filled ≥ 1 benzodiazepine prescription (IA-free controls: 22.5%, p-value < 0.001).

**Table 2:** Mean number of antidepressant/anxiolytic prescriptions dispensed and mental health services accessed by individuals with inflammatory arthritis (IA) and IA-free controls in the first year after diagnosis of incident depression or anxiety

	Depression			Anxiety		
	IA mean (SD)	IA-free controls mean (SD)	*p*-value	IA mean (SD)	IA-free controls mean (SD)	*p*-value
**Pharmacotherapy**						
**Antidepressants**						
Total antidepressant prescriptions	5.38 (12.02)	5.02 (12.58)	**< 0.001**	6.11 (15.55)	4.68 (11.20)	**< 0.001**
Days’ supply^A^	226.3 (127.8)	223.8 (127.7)	0.166	239.6 (128.5)	236.5 (125.7)	0.076
Proportion of days covered	0.62 (0.35)	0.61 (0.35)	0.166	0.66 (0.35)	0.65 (0.34)	0.076
**Anxiolytics**						
Total anxiolytic prescriptions	1.39 (4.77)	1.00 (3.85)	**< 0.001**	3.37 (8.90)	2.16 (5.85)	**< 0.001**
Days’ supply (benzodiazepines)^A, B^	112.7 (123.1)	97.5 (115.6)	**< 0.001**	123.7 (127.5)	112.4 (125.3)	**0.003**
Proportion of days covered (benzodiazepines)^B^	0.31 (0.34)	0.27 (0.32)	**< 0.001**	0.34 (0.35)	0.31 (0.34)	**0.003**
**Psychological Treatment**						
**Outpatient visits**						
All mental health services	2.60 (5.51)	2.83 (6.81)	0.531	2.97 (6.99)	2.98 (7.99)	0.427
**By service type**						
Psychiatrist	0.32 (1.67)	0.39 (2.29)	0.106	0.34 (1.87)	0.34 (2.06)	0.495
Publicly funded counselling	1.19 (1.46)	1.16 (1.42)	0.521	0.97 (1.38)	0.97 (1.38)	0.574
Publicly funded psychotherapy	1.05 (4.56)	1.25 (5.63)	0.053	1.63 (5.89)	1.65 (6.82)	0.058
Telehealth	0.00 (0.23)	0.01 (0.40)	0.655	0.00 (0.07)	0.01 (0.32)	0.591
Mental health planning	0.04 (0.34)	0.04 (0.35)	0.096	0.04 (0.37)	0.03 (0.35)	0.381
**Inpatient visits**						
All-cause hospitalization	0.65 (1.33)	0.51 (1.39)	**< 0.001**	0.84 (1.70)	0.65 (1.38)	**< 0.001**
Hospitalization for depression	0.12 (0.63)	0.11 (1.00)	**0.009**	0.16 (1.08)	0.11 (0.54)	**0.001**
Hospitalization for anxiety	0.02 (1.18)	0.02 (0.15)	0.776	0.21 (0.49)	0.17 (0.45)	**< 0.001**
Hospitalization for depression and anxiety	0.01 (0.15)	0.01 (0.12)	0.581	0.10 (0.36)	0.07 (0.29)	**0.002**

^A^Days’ supply refers to the mean days of supply of antidepressant/anxiolytic prescriptions dispensed over 365 days for IA and IA-free controls with at least one antidepressant/anxiolytic dispensed.

^B^Analyses restricted to benzodiazepines only as these were the most frequently prescribed anxiolytics.

*Abbreviations: IA – inflammatory arthritis; SD – standard deviation*

**Table 3:** Proportion of individuals with inflammatory arthritis (IA) and IA-free controls with ≥ 1 antidepressant/anxiolytic prescription dispensed and ≥ 1 mental health service accessed in the first year after diagnosis of incident depression or anxiety

	Depression			Anxiety		
	IA *n* (%)	IA-free controls *n* (%)	*p*-value	IA *n* (%)	IA-free controls *n* (%)	*p*-value
**Pharmacotherapy**						
**Antidepressants**						
All antidepressants	3963 (64.3)	3829 (61.4)	**0.001**	1911 (58.5)	1805 (54.2)	**< 0.001**
**By class**						
Selective serotonin reuptake inhibitors	2610 (42.3)	2666 (42.7)	0.650	1090 (33.4)	1139 (34.2)	0.471
Tricyclic antidepressants	666 (10.8)	442 (7.1)	**< 0.001**	418 (12.8)	259 (7.8)	**< 0.001**
Other^A^	1712 (27.8)	1538 (24.7)	**< 0.001**	1013 (31.0)	837 (25.1)	**< 0.001**
**By prescriber**						
Family physician-prescribed	3747 (60.8)	3624 (58.1)	**0.002**	1772 (54.3)	1661 (49.9)	**< 0.001**
Psychiatrist-prescribed	470 (7.6)	523 (8.4)	0.119	336 (10.3)	318 (9.6)	0.318
Rheumatologist-prescribed	50 (0.8)	< 5^B^	**< 0.001**	21 (0.6)	< 5^B^	**< 0.001**
**Anxiolytics**						
All anxiolytics	1700 (27.6)	1474 (23.6)	**< 0.001**	1529 (46.8)	1336 (40.1)	**< 0.001**
**By class**						
Benzodiazepines	1605 (26.0)	1402 (22.5)	**< 0.001**	1470 (45.0)	1298 (39.0)	**< 0.001**
Hydroxyzine	112 (1.8)	88 (1.4)	0.073	75 (2.3)	39 (1.2)	**0.001**
Buspirone	23 (0.4)	22 (0.4)	0.850	59 (1.8)	53 (1.6)	0.500
**By prescriber**						
Family physician-prescribed	1586 (25.7)	1344 (21.5)	**< 0.001**	1433 (43.9)	1234 (37.1)	**< 0.001**
Psychiatrist-prescribed	120 (1.9)	126 (2.0)	0.769	151 (4.6)	144 (4.3)	0.559
Rheumatologist-prescribed	13 (0.2)	0 (0.0)	**< 0.001**	7 (0.2)	0 (0.0)	**0.007**
**Psychological Treatment**						
**Outpatient visits**						
All mental health services	4110 (66.7)	4164 (66.7)	0.919	1905 (58.3)	1893 (56.9)	0.229
**By service type**						
Psychiatrist	840 (13.6)	909 (14.6)	0.130	432 (13.2)	424 (12.7)	0.553
Publicly funded counselling	3421 (55.5)	3456 (55.4)	0.921	1555 (47.6)	1545 (46.4)	0.328
Publicly funded psychotherapy	996 (16.2)	1086 (17.4)	0.062	614 (18.8)	562 (16.9)	**0.042**
Telehealth	8 (0.1)	10 (0.2)	0.655	9 (0.3)	7 (0.2)	0.590
Mental health planning	130 (2.1)	106 (1.7)	0.095	58 (1.8)	50 (1.5)	0.381
**Inpatient visits**						
All-cause hospitalization	2165 (35.1)	1810 (29.0)	**< 0.001**	1368 (41.9)	1130 (33.9)	**< 0.001**
Hospitalization for depression	564 (9.1)	488 (7.8)	**0.008**	323 (9.9)	251 (7.5)	**0.001**
Hospitalization for anxiety	90 (1.5)	95 (1.5)	0.772	585 (17.9)	487 (14.6)	**< 0.001**
Hospitalization for depression and anxiety	61 (1.0)	68 (1.1)	0.581	268 (8.2)	207 (6.2)	**0.002**

^A^Other antidepressants included selective serotonin-norepinephrine reuptake inhibitors, trazodone, and mirtazapine.

^B^Cell sizes < 5 not reported according to agreements of the data access request.

*Abbreviations: IA – inflammatory arthritis*

#### Psychological treatment

IA and IA-free controls with depression did not significantly differ in the overall receipt of psychological treatment (Tables 2 and 3). The proportion of individuals with depression having ≥ 1 all-cause hospitalization (IA: 35.1%, IA-free controls: 29.0%, p-value < 0.001) or ≥ 1 hospitalization for depression (IA: 9.1%, IA-free controls: 7.8%, p-value = 0.008) was significantly higher for individuals with IA.

#### Minimally adequate pharmacotherapy and/or psychological treatment

The receipt of minimally adequate pharmacotherapy for depression was observed in 50.5% (3113/6166) of those with IA and 48.0% (2993/6239) of IA-free controls. Minimally adequate psychological treatment was observed among 19.6% (1208/6166) of individuals with IA and 19.7% (1231/6239) of IA-free controls. The overall receipt of minimally adequate treatment (pharmacotherapy or psychotherapy) occurred in 59.2% (3650/6166) of individuals with IA and 56.7% (3538/6239) of IA-free controls. Among individuals with IA, females had a greater tendency to receive minimally adequate pharmacotherapy (females 51.8%; males 48.0%; chi-square p-value = 0.005), while a higher proportion of males received minimally adequate psychological treatment (females 18.2%; males 22.3%; chi-square p-value = < 0.001). Multivariable logistic regression models indicate no meaningful difference between IA and IA-free controls with respect to minimally adequate mental health treatment for depression (Table 4). Stratification by sex and type of IA (AS, PsA, and RA) produced similar findings (Supplementary Tables 3 and 4).

**Table 4:** Odds of receiving minimally adequate pharmacotherapy, psychological treatment, and pharmacotherapy or psychological treatment for depression and anxiety in individuals with inflammatory arthritis (IA) compared to IA-free controls

	Depression OR (95% CI)	Anxiety OR (95% CI)
**1: Minimally adequate pharmacotherapy**		
Unadjusted model		
	1.11 (1.03, 1.19)	1.12 (1.02, 1.23)
**Adjusted model** ^**A**^		
	1.09 (1.02, 1.17)	1.10 (1.00, 1.21)
**2: Minimally adequate psychological treatment**		
**Unadjusted model**		
	0.99 (0.91, 1.08)	1.08 (0.96, 1.22)
**Adjusted model** ^**A**^		
	0.99 (0.90, 1.08)	1.07 (0.94, 1.21)
**3: Minimally adequate pharmacotherapy or psychological treatment**		
**Unadjusted model**		
	1.11 (1.03, 1.19)	1.09 (0.99, 1.20)
**Adjusted model** ^**A**^		
	1.09 (1.02, 1.18)	1.07 (0.97, 1.18)

^A^Adjusted for age, sex, Charlson-Romano comorbidity index, neighbourhood income quintile, and residence.

*Abbreviations: 95% CI – 95% confidence interval; OR – odds ratio*

### Anxiety

#### Pharmacotherapy

Analysis by drug class highlights significant differences in the treatment of anxiety regarding the proportion with ≥ 1 tricyclic antidepressant (IA: 12.8%, IA-free controls: 7.8%, p-value < 0.001), other antidepressant (IA: 31.0%, IA-free controls: 25.1%, p-value < 0.001) and benzodiazepine derivative (IA: 45.0%, IA-free controls: 39.0%, p-value < 0.001) dispensed by IA and IA-free controls (Table 3). Days’ supply of benzodiazepines was greater among individuals with IA (IA: 123.7 ± 127.5, IA-free controls: 112.4 ± 125.3, p-value = 0.003) (Table 2).

#### Psychological treatment

The receipt of outpatient mental health services did not differ between IA and IA-free controls within the 12-months after a diagnosis of anxiety (Tables 2 and 3). However, the proportion of individuals with anxiety having ≥ 1 mental health or all-cause hospitalization was higher among individuals with IA compared to IA-free controls (Table 3).

#### Minimally adequate pharmacotherapy and/or psychological treatment

Minimally adequate pharmacotherapy for anxiety was observed in 46.9% (1530/3266) of individuals with IA and 44.1% (1467/3329) of IA-free controls. Fewer individuals received minimally adequate psychological treatment, specifically 20.2% (660/3266) of IA and 19.0% (633/3329) of IA-free controls. The receipt of minimally adequate treatment for anxiety, either pharmacotherapy or psychological treatment, was observed in 53.3% (1740/3266) of IA and 51.1% (1701/3329) of IA-free controls. Evaluation of sex differences among individuals with IA indicated a higher proportion of females received minimally adequate pharmacotherapy (females 48.1%; males 41.1%; chi-square p-value = < 0.001) and no difference regarding adequate psychological therapy (females 19.5%; males 22.3%; chi-square p-value = 0.081). Multivariable logistic regression models suggest no significant difference between IA and IA-free controls with respect to the receipt of minimally adequate pharmacotherapy and psychological treatment for incident anxiety (Table 4). Multivariable models stratified by sex and type of IA did not diverge from the primary analysis, apart from individuals with PsA having significantly higher odds of receiving minimally adequate psychological treatment for incident anxiety in comparison to IA-free controls (adjusted OR 1.62, 95% CI 1.08 to 2.43) (Supplementary Tables 3 and 4).

### Depression and anxiety

Incident comorbid depression and anxiety was identified in 861 individuals with IA (Supplementary Table 5). The overall receipt of minimally adequate treatment (pharmacotherapy or psychological) was 63.3% for IA, and 64.8% for IA-free controls. Details on pharmacotherapy and psychological treatments are presented in Supplementary Tables 6 and 7.

## Discussion

Our population-based study evaluating the receipt of minimally adequate treatment for incident depression and anxiety in individuals with IA emphasizes substantial undertreatment, with approximately only half receiving ≥ 84 days’ supply of antidepressants and one-fifth receiving ≥ 4 mental health services. Results of this study also suggest that the odds of receiving minimally adequate treatment by means of antidepressants and/or psychological treatments is similar between individuals with IA and IA-free controls. Differences in the treatment of depression and anxiety in persons with IA relative to the general population emerge when evaluating the receipt of specific pharmacological classes of antidepressants (e.g., TCA’s) and anxiolytics (e.g., benzodiazepine derivatives), as well as all-cause and mental health related hospitalizations. Given the scarcity of literature surrounding the treatment of comorbid mental health conditions in individuals with IA, the findings of our study provide detailed insight into the treatment of incident depression and anxiety that can be harnessed to improve the delivery of mental health care.

Prompt and effective treatment of comorbid depression is important given both general and IA-specific health consequences, including delayed IA remission, increased risk of mortality, and as demonstrated in our present study, greater all-cause hospitalizations [29–33]. Evaluation of the first 12-months after an incident depression diagnosis indicated that 59.2% of individuals with IA received minimally adequate pharmacotherapy or psychological treatment, a proportion similar to IA-free controls (56.7%). Although our analysis indicated a significantly higher proportion of individuals with IA were dispensed ≥ 1 antidepressant prescription (IA: 64.3%, IA-free controls: 61.2%, p-value = 0.001), the average days’ supply of antidepressants was similar between IA (226 days) and IA-free controls (224 days, p-value = 0.166). In the absence of similar analyses in IA, an assessment of the treatment of prevalent depression in the context of other chronic diseases (e.g., diabetes, asthma) noted a greater tendency for persons with chronic diseases to receive ≥ 1 counselling/psychotherapy session, but not antidepressant therapy, relative to controls [34]. Our study provides reassurance that IA diagnosis does not negatively impact the receipt of pharmacotherapy or psychological therapy for incident depression compared to IA-free controls, whilst highlighting that a substantial proportion of patients remain undertreated within the publicly funded health system.

The treatment of anxiety disorders is dependent on the specific diagnosis and includes psychological treatments as well as pharmacotherapies primarily in the form of antidepressants, with anxiolytics (i.e., benzodiazepines) intended to serve as a short-term adjunctive therapy [27]. There has been considerably less focus on the epidemiology and treatment of anxiety compared to depression in IA, despite being a leading mental disorder. Therefore, our study, which illustrates 46.9% of individuals with IA compared received minimally adequate pharmacotherapy and 20.2% received adequate psychological treatment in the 12-months after incident anxiety, is an important contribution to the literature. Moreover, our findings provide insight into the repercussions of anxiety among people with IA, given that relative to IA-free controls a greater proportion of the IA cohort experienced all-cause hospitalizations and hospitalizations specifically for mental health. Greater hospitalization for mental disorders among those with IA may arise from several factors that necessitate future investigation, such as an overall increased severity of anxiety in the IA population relative to the general population, delays in diagnosis and/or treatment of anxiety for those with IA, and the role of systemic and personal barriers in accessing mental health care.

Opportunities to support rheumatology patients with their mental health include education on how depression and anxiety are common comorbidities that accompany IA and directing patients to available resources. Unfortunately, once patients are ready to engage in help seeking for their mental health, they face systemic barriers to accessing adequate care. These barriers include scarcity of publicly funded services and financial obstacles for accessing private services. Expansion of the publicly funded health care systems to include services from mental health professionals (e.g., psychologist, licensed counselor) is ideal, yet remains an arduous task. Therefore, it is also fundamental to explore ways to increase the accessibility of mental health treatments for rheumatology patients. For instance, supported self-management, peer support groups, and IA-specific programs, such as a self-guided online cognitive behavioural therapy intervention for RA that has demonstrated reduced depression and anxiety symptoms at 3-month follow-up [35]. On the other hand, the uptake of pharmacological treatment in the form of antidepressants may be influenced severity of depression and/or anxiety, prior treatment choices, presence of comorbidities, and patient preference [24, 27]. A patients’ engagement with treatment may display heterogeneity by sex and gender, and our stratified analyses indeed illustrates a greater proportion of females with IA received adequate pharmacotherapy (females 51.8%; males 48.0%) for depression, while males were more likely to have adequate psychotherapy (females 18.2%; males 22.3%). Physicians’ training and practice should therefore be informed by known gender differences when discussing and implementing treatment options with patients [36, 37]. Moreover, qualitative work by Machin et al. [38] has outlined additional barriers to accessing care for depression and/or anxiety specific to individuals with RA, namely, physician focus on physical over mental health, stigma, poor continuity of care, limited appointment duration, and concern over medication interactions. The latter could potentially benefit from in-depth clinical counseling on drug interactions and inclusion of pharmacists during the initiation phase of an antidepressant.

Analysis of treatment patterns by pharmaceutical class identified that individuals with IA had a significantly greater likelihood of dispensing at least one prescription for tricyclic antidepressants (TCAs) and dispensing at least one prescription for and a higher days’ supply of benzodiazepines. When pharmacotherapy is indicated and aligns with patients’ preferences, first-line therapy for depression and anxiety is generally antidepressants in the form of selective serotonin reuptake inhibitors (SSRIs) and serotonin and noradrenaline reuptake inhibitors (SNRIs) [24, 27]. This is reflected in our results as a high proportion of IA and IA-free individuals initiated treatment with second generation antidepressants. However, the greater proportion of individuals with IA being prescribed TCAs may be influenced by off-label uses of antidepressants. Wong et al. [39] used electronic medical records from 2006 to 2015 in Quebec, Canada to assess treatment indications for antidepressants and while depression (55.2%) and anxiety (18.5%) were the predominant indications, insomnia (10.2%) and pain (6.1%) accounted for many off-label antidepressant prescriptions. Indeed, TCAs constituted 74.1% of antidepressant prescriptions for pain and 21.7% for insomnia [39]. In addition, although benzodiazepines are an approved short-term adjunctive therapy for anxiety disorders, they are classified as a second-line therapy with the purpose of providing symptom relief during acute crises and until antidepressants reach full therapeutic effect [27]. Long-term use is not advised given the risk of adverse events (e.g., cognitive impairment) and dependency [27]. We cannot infer consumption patterns of benzodiazepines after dispensation or off-label prescribing indications (e.g., sleep, muscle pain) with our data source, nonetheless, our findings point to the importance of medication management. Pharmacists are particularly suited to support patients with IA and comorbid mental disorders, as they can provide medication reviews to identify inappropriate prescribing and address barriers to initiating or continuing pharmacotherapy, such as continuity of care and concerns about drug interactions.

There are several limitations to our study given the nature of the data source. A major consideration is our administrative health databases do not capture privately delivered mental health services (e.g., counselors, psychologists). Therefore, our findings likely underestimate the receipt of minimally adequate psychological therapy. For context, evidence from the Canadian Community Health Survey-Mental Health notes a similar proportion of individuals with arthritis (21.1%) and without arthritis (20.9%) access counselling or psychotherapy for mood and anxiety disorders [40]. The severity of depression and anxiety will also influence the treatment approach, for example, first-line treatments for mild cases of major depression are psychological treatment, education, and/or self-management [24]. Unfortunately, ICD-9/10 coding at the level that designates severity is inaccurate, thereby preventing analyses stratified by severity of depression or anxiety [41]. Several social determinants of health associated with the diagnosis and treatment of mental disorders are also unfortunately not captured in our data source. Last, the PharmaNet database used to evaluate pharmacotherapy specifically reports dispensed prescriptions and does not capture patient refusal of medications, unfilled prescriptions, and use of medications after dispensation.

## Conclusions

As the first population-based study on the treatment of incident depression and anxiety in individuals with IA, our results emphasize that a substantial proportion fall below the threshold for minimally adequate pharmacotherapy and/or psychological treatment. We also highlight opportunities to optimize pharmacotherapy for those with IA and comorbid depression and anxiety through the monitoring of off-label prescriptions for TCAs and benzodiazepines. Future studies should focus on patients’ response to mental health treatments as well as ongoing treatment during the maintenance phase for depression and anxiety, given that physical conditions such as IA increase the risk of chronicity and recurrence of these mental disorders.

## Electronic supplementary material

Below is the link to the electronic supplementary material.

Supplementary Material 1

## Data Availability

The data that support the findings of this study are available from Population Data BC, but restrictions apply to the availability of these data, which were used under license for the current study, and so are not publicly available. Access to data provided by the Data Steward(s) is subject to approval but can be requested for research projects through the Data Steward(s) or their designated service providers.

## Abbreviations

95% CI: 95% confidence interval
AS: Ankylosing spondylitis
IA: Inflammatory arthritis
OR: Odds ratio
PsA: Psoriatic Arthritis
RA: Rheumatoid Arthritis
SNRI: Serotonin and noradrenaline reuptake inhibitors
SSRI: Selective serotonin reuptake inhibitors
TCA: Tricyclic antidepressant
