# Supplementary material for: Do individuals with inflammatory arthritis receive minimally adequate treatment for incident depression and anxiety: A population-based study
Source: Arthritis Res Ther. 2025 Jan 21;27:13. doi: 10.1186/s13075-024-03466-8 (PMC11748246; doi:10.1186/s13075-024-03466-8)
Supplement: Supplementary file 1 — Supplementary Material 1 [file 13075_2024_3466_MOESM1_ESM.docx]

**SUPPLEMENTARY MATERIALS**

**Supplementary Figure 1.** Overview of data sources, source population, and study sample for individuals with inflammatory arthritis (IA) and IA-free controls with incident depression and/or anxiety (dashed arrow shows linkages between databases using provincial health numbers which are then de-identified/scrambled).
*Abbreviations: DIN/PIN – drug identification number/product identification number; PHN – provincial health number.*

| **Supplementary Table 1.** Medication codes for antidepressants and anxiolytic according to the Anatomical Therapeutic Chemical (ATC) classification system. | |
| --- | --- |
| **ATC Classification** | **Chemical Class** |
| **N06A** | **Antidepressants** |
| N06AA | Non-selective monoamine reuptake inhibitors |
| N06AB | Selective serotonin reuptake inhibitors |
| N06AF | Monoamine oxidase inhibitors, non-selective |
| N06AG | Monoamine oxidase A inhibitors |
| N06AX | Other antidepressants |
| **NO5B** | **Anxiolytics** |
| N05BA | Benzodiazepine derivatives |
| N05BB | Diphenylmethane derivatives |
| N05BC | Carbamates |
| N05BD | Dibenzo-bicyclo-octadiene derivatives |
| N05BE | Azaspirodecanedione derivatives |
| N05BX | Other anxiolytics |

| **Supplementary Table 2.** Procedural codes for psychological treatments (counselling, psychotherapy, telehealth, psychiatry, and general practitioner) | |
| --- | --- |
| **Fee item code(s)** | **Definition** |
| **Counselling** | |
| 12120, 00120, 15320, 16120, 17120, 18120 | Individual counselling – in office |
| 12220, 13220, 15220, 16220, 17220, 18220 | Individual counselling – out of office |
| 00121, 00122, 00513, 00515 | Group counselling |
| 00514 | Prolonged visit for counselling |
| 00276, 00277 | Prolonged visit/or counselling complex case |
| **Psychotherapy** | |
| 00630, 00631, 00632 | Individual psychotherapy (office or hospital out-patient) |
| 00650, 00651, 00652 | Individual psychotherapy (hospital or institution in-patient or home) |
| 00633, 00635, 00636, 00638, 00639 | Family/Conjoint Psychotherapy |
| 00663, 00664, 00665, 00666, 00667, 00668, 00669, 00670, 00671, 00672, 00673, 00674, 00675, 00676, 00677, 00678, 00679, 00680, 00681 | Group Psychotherapy |
| **Telehealth** | |
| 13018 | Telehealth GP in-office individual counselling |
| 13021, 13022 | Telehealth GP out-of-office group counselling |
| 13038 | Telehealth GP in-office individual counselling for prolonged visit |
| 60607 | Telehealth subsequent office visit – psychiatric treatment |
| 60608 | Telehealth hospital in-patient visit – psychiatric treatment |
| 60610 | Full telehealth consultation – psychiatry |
| 60613 | Telehealth geriatric consult – psychiatry |
| 60625 | Telehealth repeat or limited consult – psychiatry |
| 60630, 60631, 60632 | Individual telehealth psychiatric treatment |
| **Psychiatrist visits** | |
| 00605 | Emergency visit – psychiatry |
| 00607 | Office visit – psychiatry |
| 00608 | Hospital visit – psychiatry |
| 00609 | Home visit – psychiatry |
| 00610 | Individual consultation – psychiatry |
| 00625 | Individual repeat consultation – psychiatry |
| 00699 | Miscellaneous – psychiatry |
| **GP mental health planning** | |
| 14044, 14045, 14046, 14047, 14048 | GP Mental health management |

| **Supplementary Table 3.** Sex-stratified odds of receiving minimally adequate pharmacotherapy, psychological treatment, and pharmacotherapy or psychological treatment for depression and anxiety in individuals with inflammatory arthritis (IA) compared to IA-free controls. | | | | |
| --- | --- | --- | --- | --- |
|  | **Depression**  **OR (95% CI)** | | **Anxiety**  **OR (95% CI)** | |
|  | **Males** | **Females** | **Males** | **Females** |
| **1: Minimally adequate pharmacotherapy** | | | | |
| **Unadjusted model** | | | | |
|  | 1.07 (0.95, 1.21) | 1.12 (1.03, 1.22) | 1.14 (0.93, 1.38) | 1.11 (1.00, 1.25) |
| **Adjusted model^A^** | | | | |
|  | 1.06 (0.94, 1.20) | 1.11 (1.02, 1.21) | 1.14 (0.93, 1.38) | 1.10 (0.98, 1.23) |
| **2: Minimally adequate psychological treatment** | | | | |
| **Unadjusted model** | | | | |
|  | 1.01 (0.87, 1.17) | 0.98 (0.88, 1.09) | 1.10 (0.87, 1.39) | 1.07 (0.93, 1.23) |
| **Adjusted model^A^** | | | | |
|  | 1.02 (0.88, 1.18) | 0.97 (0.87, 1.09) | 1.10 (0.87, 1.39) | 1.06 (0.92, 1.22) |
| **3: Minimally adequate pharmacotherapy or psychological treatment** | | | | |
| **Unadjusted model** | | | | |
|  | 1.07 (0.95, 1.21) | 1.13 (1.03, 1.23) | 1.06 (0.88, 1.28) | 1.10 (0.99, 1.23) |
| **Adjusted model^A^** | | | | |
|  | 1.06 (0.94, 1.20) | 1.11 (1.02, 1.21) | 1.05 (0.87, 1.28) | 1.09 (0.97, 1.22) |
| ^A^Adjusted for age, Charlson-Romano comorbidity index, neighbourhood income quintile, and residence.  *Abbreviations: 95% CI – 95% confidence interval; OR – odds ratio* | | | | |

| **Supplementary Table 4.** Inflammatory arthritis (IA)-stratified odds of receiving minimally adequate pharmacotherapy, psychological treatment, and pharmacotherapy or psychological treatment for depression and anxiety in individuals with IA compared to IA-free controls. | | | | | | |
| --- | --- | --- | --- | --- | --- | --- |
|  | **Depression**  **OR (95% CI)** | | | **Anxiety**  **OR (95% CI)** | | |
|  | **Ankylosing Spondylitis** | **Psoriatic Arthritis** | **Rheumatoid Arthritis** | **Ankylosing Spondylitis** | **Psoriatic Arthritis** | **Rheumatoid Arthritis** |
| **1: Minimally adequate pharmacotherapy** | | | | | | |
| **Unadjusted model** | | | | | | |
|  | 1.20 (0.97, 1.47) | 1.18 (0.91, 1.52) | 1.09 (1.01, 1.18) | 1.24 (0.94, 1.63) | 1.34 (0.96, 1.87) | 1.08 (0.97, 1.20) |
| **Adjusted model^A^** | | | | | | |
|  | 1.20 (0.98, 1.48) | 1.13 (0.87, 1.47) | 1.07 (0.99, 1.16) | 1.25 (0.94, 1.65) | 1.33 (0.95, 1.86) | 1.06 (0.95, 1.18) |
| **2: Minimally adequate psychological treatment** | | | | | | |
| **Unadjusted model** | | | | | | |
|  | 1.22 (0.95, 1.57) | 0.94 (0.69, 1.28) | 0.97 (0.87, 1.07) | 1.22 (0.87, 1.70) | 1.58 (1.06, 2.37) | 1.01 (0.88, 1.16) |
| **Adjusted model^A^** | | | | | | |
|  | 1.22 (0.94, 1.58) | 0.91 (0.67, 1.25) | 0.96 (0.87, 1.07) | 1.22 (0.87, 1.71) | 1.62 (1.08, 2.43) | 0.99 (0.86, 1.14) |
| **3: Minimally adequate pharmacotherapy or psychological treatment** | | | | | | |
| **Unadjusted model** | | | | | | |
|  | 1.23 (1.00, 1.52) | 1.15 (0.88, 1.50) | 1.09 (1.00, 1.18) | 1.23 (0.93, 1.63) | 1.27 (0.91, 1.76) | 1.05 (0.95, 1.17) |
| **Adjusted model^A^** | | | | | | |
|  | 1.23 (0.99, 1.51) | 1.09 (0.84, 1.43) | 1.07 (0.99, 1.16) | 1.23 (0.93, 1.63) | 1.27 (0.91, 1.77) | 1.03 (0.92, 1.15) |
| ^A^Adjusted for age, sex, Charlson-Romano comorbidity index, neighbourhood income quintile, and residence.  *Abbreviations: 95% CI – 95% confidence interval; OR – odds ratio* | | | | | | |

| **Supplementary Table 5.** Characteristics of individuals with inflammatory arthritis (IA) and IA-free controls with incident depression and anxiety. | | |
| --- | --- | --- |
|  | **Depression & Anxiety** | |
| **Characteristic** | **IA**  **(n=861)** | **IA-free**  **Controls**  **(n=861)** |
| Age, mean (SD) | 52.0 (18.8) | 51.9 (18.8) |
| Female, n (%) | 608 (70.6) | 608 (70.6) |
| Type of inflammatory arthritis, n (%) |  |  |
| Ankylosing spondylitis | 96 (11.2) | --- |
| Psoriatic arthritis | 70 (8.1) | --- |
| Rheumatoid arthritis | 695 (80.7) | --- |
| Charlson-Romano comorbidity index, mean (SD) | 0.92 (1.04) | 0.32 (0.89) |
| Neighbourhood income quintile, n (%) |  |  |
| Quintile 1 | 199 (23.1) | 170 (19.7) |
| Quintile 2 | 200 (23.2) | 167 (19.4) |
| Quintile 3 | 141 (16.4) | 194 (22.5) |
| Quintile 4 | 180 (20.9) | 161 (18.7) |
| Quintile 5 | 141 (16.4) | 169 (19.6) |
| Residence, n (%) |  |  |
| Urban | 144 (16.7) | 743 (86.3) |
| Rural | 717 (83.3) | 118 (13.7) |
| Descriptive statistics were determined for the year prior to IA index date.  *Abbreviations: IA – inflammatory arthritis; SD – standard deviation* | | |

| **Supplementary Table 6.** Mean number of antidepressant/anxiolytic prescriptions dispensed, and mental health services accessed by individuals with inflammatory arthritis (IA) and IA-free controls in the first year after diagnosis of incident depression and anxiety. | | | |
| --- | --- | --- | --- |
|  | **Depression & Anxiety** | | |
|  | **IA**  **mean (SD)** | **IA-free controls**  **mean (SD)** | **p-value** |
| **Pharmacotherapy** | | | |
| **Antidepressants** | | | |
| Total antidepressant prescriptions | 7.42 (19.64) | 5.92 (9.42) | 0.987 |
| Days’ supply^A^ | 234.5 (126.3) | 247.5 (121.8) | 0.076 |
| Proportion of days covered | 0.64 (0.35) | 0.68 (0.33) | 0.076 |
| **Anxiolytics** | | | |
| Total anxiolytic prescriptions | 3.03 (7.49) | 2.65 (6.76) | 0.576 |
| Days’ supply (benzodiazepines)^A, B^ | 114.8 (125.2) | 110.4 (121.8) | 0.797 |
| Proportion of days covered (benzodiazepines)^B^ | 0.31 (0.34) | 0.30 (0.33) | 0.797 |
| **Psychological Treatment** | | | |
| **Outpatient visits** | | | |
| All mental health services | 3.55 (7.68) | 4.34 (11.20) | 0.242 |
| **By service type** |  |  |  |
| Psychiatrist | 0.47 (1.86) | 0.61 (2.81) | 0.446 |
| Publicly funded counselling | 1.03 (1.43) | 1.00 (1.38) | 0.536 |
| Publicly funded psychotherapy | 2.00 (6.47) | 2.74 (9.60) | 0.087 |
| Telehealth | 0.01 (0.11) | 0.00 (0.04) | 0.315 |
| Mental health planning | 0.07 (0.46) | 0.05 (0.35) | 0.285 |
| **Inpatient visits** | | | |
| All-cause hospitalization | 0.99 (2.48) | 0.74 (1.50) | **0.019** |
| Hospitalization for depression | 0.29 (1.98) | 0.21 (0.96) | **0.039** |
| Hospitalization for anxiety | 0.24 (0.58) | 0.20 (0.46) | 0.262 |
| Hospitalization for depression and anxiety | 0.15 (0.48) | 0.11 (0.36) | **0.025** |
| ^A^Days’ supply refers to the mean days of supply of antidepressant/anxiolytic prescriptions dispensed over 365 days for IA and IA-free controls with at least one antidepressant/anxiolytic dispensed.  ^B^Analyses restricted to benzodiazepines only as these were the most frequently prescribed anxiolytics.  *Abbreviations: IA – inflammatory arthritis; SD – standard deviation* | | | |

| **Supplementary Table 7.** Proportion of individuals with inflammatory arthritis (IA) and IA-free controls with ≥1 antidepressant/anxiolytic prescription dispensed and ≥1 mental health service accessed in the first year after diagnosis of incident depression and anxiety. | | | |
| --- | --- | --- | --- |
|  | **Depression & Anxiety** | | |
|  | **IA**  **n (%)** | **IA-free controls**  **n (%)** | **p-value** |
| **Pharmacotherapy** | | | |
| **Antidepressants** | | | |
| All antidepressants | 507 (68.0) | 514 (68.7) | 0.754 |
| **By class** |  |  |  |
| Selective serotonin reuptake inhibitors | 281 (37.7) | 349 (46.7) | **<0.001** |
| Tricyclic antidepressants | 82 (11.0) | 60 (8.0) | 0.050 |
| Other^A^ | 290 (38.9) | 233 (31.1) | **0.002** |
| **By prescriber** |  |  |  |
| Family physician-prescribed | 466 (62.5) | 469 (62.7) | 0.926 |
| Psychiatrist-prescribed | 101 (13.5) | 100 (13.4) | 0.923 |
| Rheumatologist-prescribed | <5^B^ | <5^B^ | 0.374 |
| **Anxiolytics** | | | |
| All anxiolytics | 344 (46.1) | 342 (45.7) | 0.880 |
| **By class** |  |  |  |
| Benzodiazepines | 330 (44.2) | 331 (44.3) | 0.995 |
| Hydroxyzine | 14 (1.9) | 12 (1.6) | 0.687 |
| Buspirone | 17 (2.3) | 10 (1.3) | 0.172 |
| **By prescriber** |  |  |  |
| Family physician-prescribed | 320 (42.9) | 311 (41.6) | 0.606 |
| Psychiatrist-prescribed | 44 (5.9) | 50 (6.7) | 0.531 |
| Rheumatologist-prescribed | <5 | 0 (0.0) | 0.062 |
| **Psychological Treatment** | | | |
| **Outpatient visits** | | | |
| All mental health services | 492 (66.0) | 456 (61.0) | **0.045** |
| **By service type** |  |  |  |
| Psychiatrist | 143 (19.2) | 130 (17.4) | 0.371 |
| Publicly funded counselling | 368 (49.3) | 344 (46.0) | 0.196 |
| Publicly funded psychotherapy | 207 (27.7) | 168 (22.5) | **0.018** |
| Telehealth | <5 | <5 | 0.374 |
| Mental health planning | 26 (3.5) | 19 (2.5) | 0.285 |
| **Inpatient visits** | | | |
| All-cause hospitalization | 319 (42.8) | 278 (37.2) | **0.027** |
| Hospitalization for depression | 125 (16.8) | 96 (12.8) | **0.033** |
| Hospitalization for anxiety | 145 (19.4) | 129 (17.2) | 0.274 |
| Hospitalization for depression and anxiety | 95 (12.7) | 68 (9.1) | **0.024** |
| ^A^Other antidepressants included selective serotonin-norepinephrine reuptake inhibitors, trazodone, and mirtazapine.  ^B^Cell sizes <5 not reported according to agreements of the data access request.  *Abbreviations: IA – inflammatory arthritis* | | | |
